# Relationship Between Fasting Blood Glucose Levels in Middle Age and Cognitive Function in Later Life: The Aichi Workers’ Cohort Study

**DOI:** 10.2188/jea.JE20210128

**Published:** 2023-02-05

**Authors:** Masako Shimoda, Kayo Kaneko, Takeshi Nakagawa, Naoko Kawano, Rei Otsuka, Atsuhiko Ota, Hisao Naito, Masaaki Matsunaga, Naohiro Ichino, Hiroya Yamada, Chifa Chiang, Yoshihisa Hirakawa, Koji Tamakoshi, Atsuko Aoyama, Hiroshi Yatsuya

**Affiliations:** 1 Department of Public Health and Health Systems, Nagoya University Graduate School of Medicine, Nagoya, Japan; 2 Department of Occupational and Environmental Health, Graduate School of Medical Sciences, Nagoya City University, Nagoya, Japan; 3 National Center for Geriatrics and Gerontology, Aichi, Japan; 4 Osaka Prefecture University, Osaka, Japan; 5 Department of Public Health, Fujita Health University School of Medicine, Aichi, Japan; 6 Department of Nursing, Nagoya University School of Health Sciences, Nagoya, Japan; 7 Nagoya University of Arts and Sciences, Aichi, Japan

**Keywords:** fasting blood glucose, cognitive assessment, middle aged, diabetes mellitus

## Abstract

**Background:**

There is limited evidence regarding the relationship between Diabetes mellitus (DM) in middle age and mild cognitive impairment after a follow-up. Therefore, we investigated the relationship between fasting blood glucose (FBG) levels in middle age and cognitive function assessed using the Japanese version of the Montreal Cognitive Assessment (MoCA-J) in later life, following over 15 years of follow-up in the Aichi Workers’ Cohort Study in Japan.

**Methods:**

Participants were 253 former local government employees aged 60–79 years in 2018 who participated in a baseline survey conducted in 2002. Using baseline FBG levels and self-reported history, participants were classified into the normal, impaired fasting glucose (IFG) and, and DM groups. Total MoCA-J score ranges from 0 to 30, and cognitive impairment was defined as MoCA-J score ≤25 in this study. A general linear model was used to estimate the mean MoCA-J scores in the FBG groups, adjusted for age, sex, educational year, smoking status, alcohol consumption, physical activity, body mass index, systolic blood pressure, total cholesterol, and estimated glomerular filtration rate.

**Results:**

The mean MoCA-J score in the total population was 25.0, and the prevalence of MoCA-J score ≤25 was 49.0%. Multivariable-adjusted total MoCA-J scores were 25.2, 24.8, and 23.4 in the normal, IFG, and DM groups, respectively. The odds ratio of MoCA-J score ≤25 in the DM group was 3.29.

**Conclusion:**

FBG level in middle age was negatively associated with total MoCA-J scores assessed later in life, independent of confounding variables.

## INTRODUCTION

The number of dementia patients is expected to reach 74.7 million worldwide in 2030 and 7.3 million in Japan, along with the increasing elderly population.^1^ Early detection of cognitive decline and possible interventions are important to prevent or delay the progression to dementia.

Mild cognitive impairment (MCI) is regarded as an intermediate stage between intact cognition and dementia. The diagnostic criteria for MCI are (1) concern regarding a change in cognition, (2) impairment in one or more cognitive domains, (3) preservation of independence in functional abilities, and (4) not demented.^2^ Those with MCI are reportedly at a high risk of transitioning to dementia within 3 years.^3^ However, not all cases of MCI progress to dementia, and some return to normal.^4^ Thus, early detection of MCI is important to prevent the transition to dementia.^5^^,^^6^

Diabetes mellitus (DM) is an established risk factor for Alzheimer’s disease and vascular dementia. In a meta-analysis using data of a study involving a 5- to 10-year follow-up of people aged 45–75 years by Xue et al,^7^ high level of fasting blood glucose (FBG) was associated with 1.2 times higher risk of all-cause dementia, compared with low FBG level. In contrast, there was no association between FBG level and the risk of developing dementia in a 15-year follow-up study of Japanese aged 60 years or older. Two other reports did not detect an association.^8^^,^^9^ Although nine studies have reported an association between DM and MCI in older people in previous cohort studies,^10^^–^^18^ evidence is limited on the relationship between DM in middle age and MCI after a follow-up period.

Moreover, long-term epidemiological studies using the Japanese version of the Montreal Cognitive Assessment (MoCA-J), which is reportedly a useful test for MCI screening, remain sparse, although a previous study in Japan examined older people for the association of DM and hypertension, using a 3-year follow-up MoCA-J performance.^19^

Therefore, we investigated the relationship between FBG levels measured in middle age and cognitive function (assessed using MoCA-J) after over 15 years of follow-up in a long-term cohort study in Japan.

## METHODS

The Aichi Workers’ Cohort Study established in 1997 is an ongoing prospective study on DM and cardiovascular diseases in Aichi Prefecture, Japan.^20^ The 2002 cohort included 6,648 civil servants aged 35–66 years who responded to a self-administered questionnaire concerning their lifestyle and medical history and provided their annual health checkup data.^21^ We obtained data including height, weight, blood pressure, FBG, total cholesterol, and creatinine from the baseline annual health checkup data.

We explained the study aims and procedures to and obtained written informed consent from all participants. The study protocol was approved by the Bioethics Review Committees of Nagoya University School of Medicine (approval number: 2007-0504) and Fujita Health University (approval number: HM18-246), Japan.

**Figure 1.**
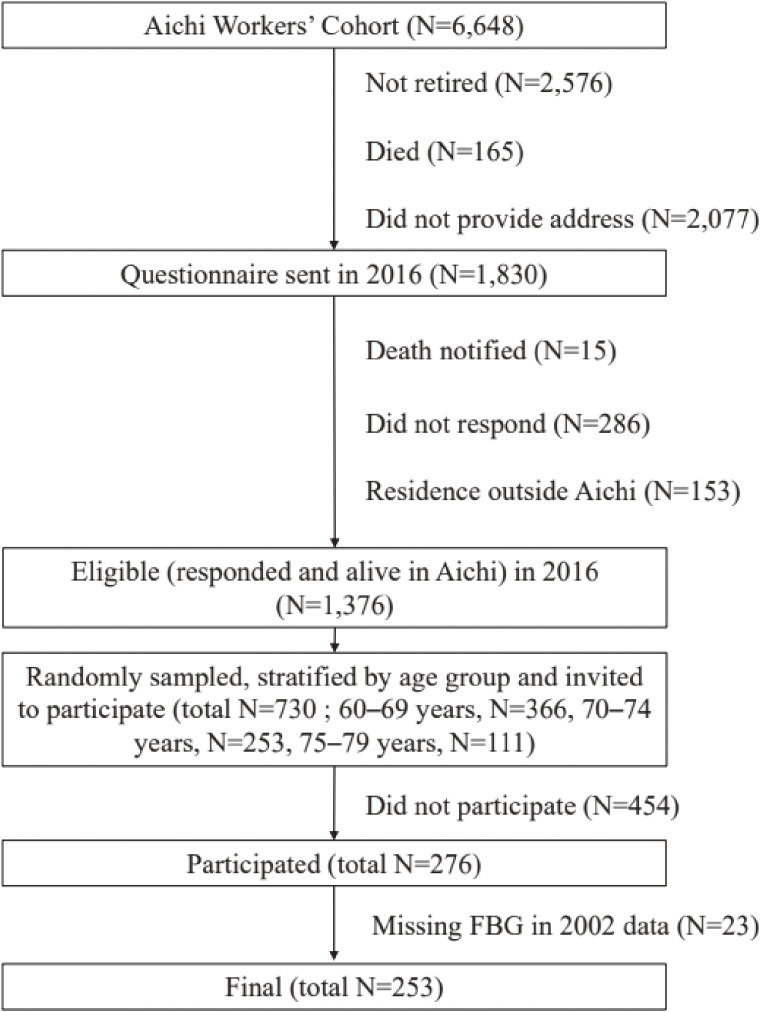
Flowchart for participant selection FBG: fasting blood glucose

In 2018, we conducted a survey of the retirees from the 2002 cohort, including cognitive function tests. From 1,376 eligible retirees, 730 people were randomly selected from three age categories: 60–69 (*n* = 366), 70–74 (*n* = 253), and 75–79 (*n* = 111) years, and 276 agreed to participate. After excluding 23 subjects with missing data on FBG in 2002, 253 subjects were finally included in this analysis (Figure 1).

The MoCA-J was performed to evaluate the cognitive function of the participants. The MoCA-J, which was developed based on the MoCA for Japanese,^22^^,^^23^ is a one-page 30-item test to be completed in 10 min. The reliability and validity of the MoCA-J for detecting early cognitive decline when compared with a conventional cognitive test were reported in a Japanese population.^22^ The reported area under the receiver operating characteristic curve for identifying MCI with MoCA-J, of 0.95, is higher than that obtained using either the Mini-Mental State Examination^24^^,^^25^ or Hasegawa’s Dementia Scale-Revised (HDS-R).^26^ Using a cut-off point of 25/26, the MoCA-J demonstrated excellent sensitivity (93.0%) and specificity (87.0%) in MCI screening. The total MoCA-J score ranges from 0 to 30; in this study, cognitive impairment was defined as a MoCA-J score ≤25.

FBG was classified into three groups: (1) normal: FBG <100 mg/dL; (2) impaired fasting glucose (IFG): FBG ≥100 and <126 mg/dL; and (3) DM: FBG ≥126 mg/dL or self-reported history of type 2 DM including the use of glucose lowering medication.

Height and weight were measured to the nearest 0.1 cm and 0.1 kg, respectively. Body mass index (BMI) was computed as body weight (kg) divided by the square of height (m^2^) and included as a continuous variable in the analyses. Estimated glomerular filtration rate (eGFR) was calculated using the Chronic Kidney Disease Epidemiology Collaboration Equation,^27^ as shown below:

(Men)

$$\begin{array}{rl} eGFR & =141\times (\text{s-Cr}/0.9{)}^{-0.411}\times 0.993^{\text{Age}},\;\\ & \;\text{for those with s-Cr }\leq 0.9\;\text{mg/dL (79.6}\;\text{µ}\text{mol/L)} \end{array}$$

or

$$\begin{array}{rl} eGFR & =141\times (\text{s-Cr}/0.9{)}^{-1.209}\times 0.993^{\text{Age}},\;\\ & \;\text{for those with s-Cr }>0.9\;\text{mg/dL (79.6}\;\text{µ}\text{mol/L)} \end{array}$$

(Women)

$$\begin{array}{rl} eGFR & =144\times (\text{s-Cr}/0.7{)}^{-0.329}\times 0.993^{\text{Age}},\;\\ & \;\text{for those with s-Cr }\leq 0.7\;\text{mg/dL (79.6}\;\text{µ}\text{mol/L)} \end{array}$$

or

$$\begin{array}{rl} eGFR & =144\times (\text{s-Cr}/0.9{)}^{-1.209}\times 0.993^{\text{Age}},\;\\ & \;\text{for those with s-Cr }>0.7\;\text{mg/dL (79.6}\;\text{µ}\text{mol/L)} \end{array}$$

(s-Cr = serum creatinine)

Smoking status was classified into three categories (never, former, and current smokers). Participants provided responses to items regarding the frequency of drinking, type of alcohol, and the amount of each type of alcohol per occasion. Alcohol consumption (g/day) was estimated based on these responses, and consumption pattern was classified into four categories (0, <23, ≥23 to <46 and ≥46 g/day). Physical activity was assessed as the number of days engaged in leisure-time physical activity for ≥60 min.

### Statistical analysis

Comparison of continuous variables among the three FBG groups was performed using analysis of variance and multiple comparison with Bonferroni methods. Comparison of categorical variables was performed using the chi-square test. Crude and adjusted means of MoCA-J scores according to the FBG categories were calculated using univariable and multivariable general linear models. *P* for linear trend was obtained using the polynomial method in the general linear model. Odds ratios (ORs) and the 95% confidence intervals (CIs) were estimated using logistic regression analysis. Model 1 was adjusted for age, sex, and educational year. Model 2 was adjusted for variables in model 1 plus smoking status (current, former, and never), alcohol consumption pattern (g/day) (0, <23, ≥23 to <46 and ≥46 g/day), and physical activity (day/week). Model 3 was adjusted for variables in model 2 plus BMI, systolic blood pressure, total cholesterol, and eGFR levels. Since a longer duration of DM is related to an increased risk of cognitive decline,^28^ we also performed stratified analysis by age group: ≤54 years and ≥55 years at baseline. We also conducted an additional analysis that defined normal, IFG, and DM groups based only on baseline glucose levels disregarding self-reported history. Statistical analysis was conducted using IBM SPSS Statistics for Windows, Version 26.0 (IBM, Armonk, NY, USA). A *P*-value <0.05 was considered significant.

## RESULTS

Table 1 shows the participants’ (253) baseline characteristics, according to the FBG categories in 2002. Of these, 180, 50, and 23 were in the normal, IFG, and DM groups, respectively. The proportion of participants were similar among the three groups with respect to the following variables: men, educational year, alcohol consumption, physical activity frequency, BMI, and total cholesterol. Age was significantly higher and the proportion of current smokers was lower in the DM group than in the other two groups, whereas systolic blood pressure and lower eGFR were significantly higher in the IFG group than in the normal group.

**Table 1.** Baseline characteristics of the participants according to fasting blood glucose levels at baseline, Aichi, 2002

	Normal (*n* = 180)	Impaired Fasting Glucose (*n* = 50)	Diabetes Mellitus (*n* = 23)	*P*-value^a^
Men, %	77.8	92.0	87.0	0.06
Age, year	53.0 (3.8)	53.7 (3.8)	55.0 (3.4)	<0.05
Education, year	15.1 (2.1)	15.4 (1.7)	14.8 (3.5)	0.51
Smoking status, %				
Current	23.5	24.0	17.4	<0.05
Former	27.4	45.0	39.1	
Never	49.1	28.0	43.5	
Alcohol consumption, %				
0 g/day	31.7	16.0	34.8	0.21
<23 g/day	41.1	44.0	30.4	
≥23 to <46 g/day	16.7	24.0	13.0	
≥46 g/day	10.6	16.0	21.7	
Physical activity, %				
≥3 days/week	23.9	18.0	30.4	0.48
Body mass index, kg/m^2^	22.8 (2.4)	23.7 (2.5)	23.5 (2.7)	0.05
Systolic blood pressure, mm Hg	126 (13)	133 (18)	130 (16)	<0.01
eGFR,^b^ mL/min/1.73 m^2^	93.4 (11.8)	86.6 (15.4)	91.6 (11.0)	<0.01
Total cholesterol, mg/dL	212 (35)	206 (38)	213 (46)	0.53

eGFR, estimated glomerular filtration rate.

Fasting blood glucose levels: normal ≤99 mg/dL; impaired fasting glucose 100–125 mg/dL; diabetes mellitus ≥126 mg/dL or self-reported type 2 diabetes mellitus.

Values are reported as mean (standard deviation or percentage).

^a^Analysis of variance and chi-square tests for continuous and categorical variables, respectively.

^b^eGFR is calculated using the Chronic Kidney Disease Epidemiology Collaboration Equation.

The prevalence of MoCA-J score ≤25 was 49.0%. Table 2 shows the mean MoCA-J score in 2018 according to the FBG categories at baseline. Model 1-adjusted total MoCA-J scores were 25.2, 24.9, and 23.5 in the normal, IFG, and DM group, respectively. The DM group had significantly lower MoCA-J scores than the normal group (*P* = 0.01, *P* for linear trend <0.01). Model 3-adjusted MoCA-J scores for the normal, IFG, and DM groups were 25.2, 24.8, and 23.4, respectively. The DM group had significantly lower MoCA-J scores than the normal group (*P* = 0.02, *P* for linear trend <0.01).

**Table 2.** Adjusted mean MoCA-J scores in 2018 according to fasting blood glucose levels at baseline, Aichi, 2002–2018

	Normal	Impaired Fasting Glucose	Diabetes Mellitus	*P*-value^a^	*P* for linear trend^b^
Model 1	25.2 (0.2)	24.9 (0.4)	23.5 (0.5)	0.01	0.003
Model 2	25.2 (0.2)	25.0 (0.4)	23.4 (0.5)	0.01	0.002
Model 3	25.2 (0.2)	24.8 (0.4)	23.4 (0.6)	0.02	0.004

MoCA-J, Japanese version of Montreal Cognitive Assessment.

Fasting blood glucose levels: normal ≤99 mg/dL; impaired fasting glucose 100–125 mg/dL; diabetes mellitus ≥126 mg/dL or self-reported type 2 diabetes mellitus.

Values are reported as mean (standard error).

Model 1: adjusted for age, sex, and education year.

Model 2: adjusted for variables in model 1 plus smoking status (current, former, and never), alcohol consumption (g/day) (0, <23, ≥23 to <46, and ≥46), physical activity (day/week).

Model 3: adjusted for variables in model 2 plus body mass index, systolic blood pressure, total cholesterol and estimated glomerular filtration rate.

^a^Analysis of covariance.

^b^Polynomial method in the general linear model.

Table 3 shows the ORs and 95% CI for MoCA-J score ≤25 according to the FBG categories. The OR for the DM group for MoCA-J score ≤25 was 3.71 (95% CI, 1.40–9.84), and in model 3, the OR for the DM group was 3.29 (95% CI, 1.10–9.80).

**Table 3.** Odds ratios and the 95% confidence intervals for declined cognitive function according to fasting blood glucose levels at baseline, Aichi, 2002–2018

	Normal	Impaired Fasting Glucose	Diabetes Mellitus
*n*/*N*	78/180	29/50	17/23

Crude	Ref	1.81 (0.96–3.41)	3.71 (1.40–9.84)
Model 1	Ref	1.60 (0.83–3.11)	2.90 (1.06–7.95)
Model 2	Ref	1.66 (0.84–3.27)	3.06 (1.10–8.46)
Model 3	Ref	2.10 (0.97–4.55)	3.29 (1.10–9.80)

*n*, number of individuals with cognitive decline; *N*, total number of individuals; Ref, reference.

Fasting blood glucose levels: normal ≤99 mg/dL; impaired fasting glucose 100–125 mg/dL; diabetes mellitus ≥126 mg/dL or self-reported type 2 diabetes mellitus.

Declined cognitive function: Japanese version of Montreal Cognitive Assessment (MoCA-J) score ≤25.

Values are reported as odds ratio (95% confidence interval).

Model 1: adjusted for age, sex, and education year.

Model 2: adjusted for variables in model 1 plus smoking status (current, former, and never), alcohol consumption (g/day) (0, <23, ≥23 to <46, and ≥46), physical activity (day/week).

Model 3: adjusted for variables in model 2 plus body mass index, systolic blood pressure, total cholesterol and estimated glomerular filtration rate.

Differences in MoCA-J scores among the three groups defined based solely on FBG levels (Normal: *n* = 186, IFG: *n* = 59, DM: *n* = 8) were also statistically significant (25.2, 24.4, and 22.8, respectively; *P* = 0.028). The associations of FBG groups with MoCA-J score ≤25 remained similar, although the confidence interval of the OR for the DM group widened significantly: 2.26 (95% CI, 1.10–4.66) in the IFG and 4.90 (95% CI, 0.51–46.87) in the DM group.

Table 4 shows the MoCA-J scores in 2018 examined per the two age groups (40–54 and ≥55 years). For age group 40–54 years, crude MoCA-J scores were 25.8, 25.2, and 24.2 in the normal, IFG, and DM groups, respectively (*P* = 0.057, *P* for linear trend <0.05). Model 3-adjusted total MoCA-J scores were 25.7, 24.7, and 24.5 in the normal, IFG, and DM groups, respectively (*P* = 0.078, *P* for linear trend = 0.095). For age group ≥55 years, crude MoCA-J scores were 24.3, 24.3, and 21.5 in the normal, IFG, and DM groups, respectively (*P* < 0.05, *P* for linear trend <0.05). Model 3-adjusted MoCA-J scores for the normal, IFG, and DM groups were 24.0, 24.4, 21.8, respectively (*P* = 0.137, *P* for linear trend = 0.072).

**Table 4.** MoCA-J scores in 2018 according to fasting blood glucose levels at baseline stratified by age group, Aichi, 2002–2018

	Normal	Impaired Fasting Glucose	Diabetes Mellitus	*P*-value^a^	*P* for linear trend^b^
40–54 years					
*N*	127	27	13		
Crude	25.8 (0.2)	25.2 (0.5)	24.2 (0.7)	0.057	0.025
Model 3	25.7 (0.2)	24.7 (0.6)	24.5 (0.7)	0.078	0.095
≥55 years					
*N*	53	23	10		
Crude	24.3 (0.5)	24.3 (0.7)	21.5 (1.0)	0.036	0.012
Model 3	24.0 (0.4)	24.4 (0.7)	21.8 (1.1)	0.137	0.072

MoCA-J, Japanese version of Montreal Cognitive Assessment.

Fasting blood glucose levels: normal ≤99 mg/dL; impaired fasting glucose 100–125 mg/dL; diabetes mellitus ≥126 mg/dL or self-reported type 2 diabetes mellitus.

Values are reported as mean (standard error); *N*, the number of individuals.

Model 3: adjusted for age, sex, educational year, smoking status (current, former, and never), alcohol consumption (g/day) (0, <23, ≥23 to <46, and ≥46), physical activity (day/week), body mass index, systolic blood pressure, total cholesterol and estimated glomerular filtration rate.

^a^Analysis of covariance.

^b^Polynomial method in the general linear model.

## DISCUSSION

We investigated the relationship between FBG categories in middle age and MCI (defined as MoCA-J ≤25) after over 15 years of follow-up in a cohort study in Japan and found that those who had DM in middle age had lower total MoCA-J scores in older age, and a higher risk of MCI than those in the normal FBG group. The present finding is consistent with that of a previous meta-analysis that showed 49% increased risk of MCI in DM, compared with that in non-DM group.^7^ Although several studies exist on the relationship between DM and MCI in the elderly, to the best of our knowledge, no studies have been conducted in middle-aged Japanese population regarding future MCI risk.

In the present study, 49.0% of participants presented with MCI assessed using MoCA-J. This MCI prevalence rate is slightly lower than that reported in a previous study (65.2%) among healthy community-dwelling older adults.^29^ The difference may be because the average age in our study (69.9 years) was less than that in the previous study (76.3 years).

As the duration of DM has been reported to affect cognitive function,^28^ we performed a stratified analysis by age. For both age groups, the results showed that people with DM had lower MoCA-J scores, suggesting that the associations did not differ by the duration of DM.

Biological mechanisms related to the association between DM and MCI include the fact that DM causes macrovascular and microvascular diseases in the brain, and cognitive decline occurs owing to blood circulation failure in the brain.^30^ Studies suggest that DM and hyperglycemia also influence the accumulation of senile plaques and neurofibrillary tangles, which are risk factors for Alzheimer’s disease.^31^^,^^32^ Furthermore, chronic hyperglycemia due to DM increases oxidative stress and damages nerve cells, possibly causing dementia.^33^

The clinical significance of the present finding may be the observed difference of 2 points in the mean MoCA-J score between the normal and DM groups, which would be equivalent to an 8-year difference according to age (from MoCA-J regression analysis result; data not shown).

Although causality could not be determined from the observational study, our findings may emphasize the importance of the prevention or control of DM in middle-aged people to prevent or delay MCI occurrence. Alternatively, those who have had DM in middle age may benefit from frequent cognitive screening, as MCI is believed to be reversible.

This study has several limitations. First, the participants, though they were retired, were sufficiently healthy to participate in the survey. They were randomly selected from the entire cohort of those who were being followed up. Second, approximately 40% of those invited actually participated in the 2018 survey. Characteristics, such as age and DM prevalence, of non-participants were similar to those of participants. Selection bias, by which participants with DM are systemically more or less likely to participate, would not be so plausible. Additionally, it would not be feasible to speculate that the association between DM and MCI differs according to participation. Third, since the number of participants, especially that of women was small, we could not present sex-stratified analysis. We, however, explored the analysis separately in men and women. The main finding that DM was associated with low MoCA-J score or higher MCI risk was similarly observed in both sexes. Fourth, although we used FBG level to determine the presence of IFG and DM, there may have been misclassification. Because DM diagnosis should also be based on other methods, participants with DM may have been included in the IFG group or non-DM participants may have been included in the DM group in this study. If non-DM participants were included in the DM group, the true relationship between MCI and DM may have been distorted. However, it is difficult to think that the misclassification of DM status occurred according to MCI. Future studies based on oral glucose tolerance test and glycated hemoglobin may be required. Finally, we used only the MoCA-J as the screening tool to determine MCI; as MCI had not been formally diagnosed, other tests might be necessary.

### Conclusion

In this study, FBG levels in middle age were shown to be independently, negatively associated with total MoCA-J scores in later life. It is important to proactively implement interventions aimed at treating and preventing DM and hyperglycemic conditions among middle-aged people to prevent MCI.
